# Supplementary figures and images for: Dengue importation into Europe: A network connectivity-based approach
Source: PLoS One. 2020 Mar 12;15(3):e0230274. doi: 10.1371/journal.pone.0230274 (PMC7067432; doi:10.1371/journal.pone.0230274)

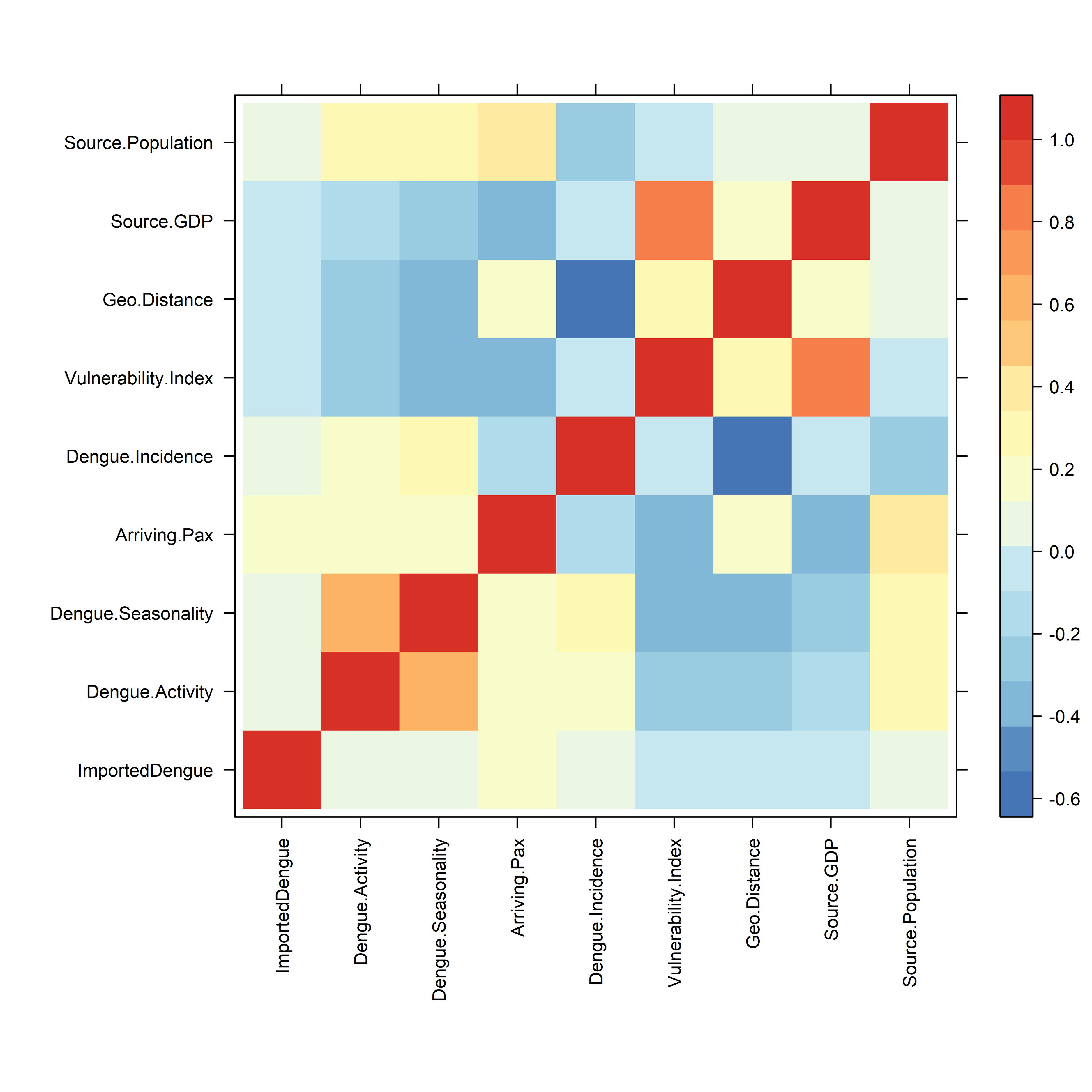

Supplement: S1 Fig — Red colors indicate strong positive correlations, dark blue indicates strong negative correlations, and yellow implies no empirical relationship between the variables. (TIF) [file pone.0230274.s001.tif]

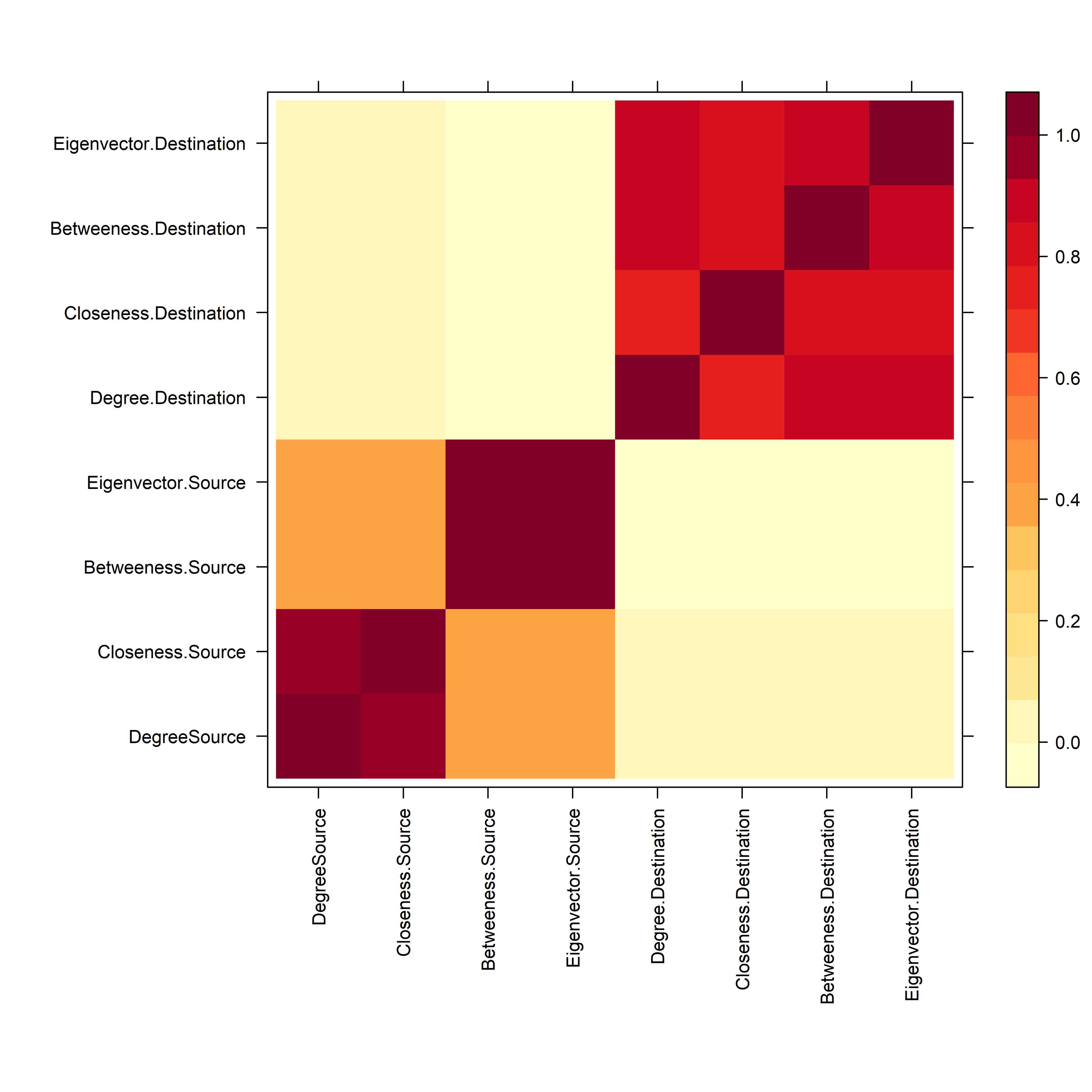

Supplement: S2 Fig — Red colors indicate strong positive correlations and yellow indicates no empirical relationship between the centrality measures. (TIF) [file pone.0230274.s002.tif]
