# Supplementary material for: Dengue importation into Europe: A network connectivity-based approach
Source: PLoS One. 2020 Mar 12;15(3):e0230274. doi: 10.1371/journal.pone.0230274 (PMC7067432; doi:10.1371/journal.pone.0230274)
Supplement: S1 Appendix — (DOCX) [file pone.0230274.s003.docx]

### **Dengue importation into Europe: a network connectivity-based approach**

Donald Salami, César Capinha, Maria do Rosário Oliveira Martins, Carla Alexandra Sousa

### **S1 Appendix: Alternative specifications of GLMM model**

## **Alternative specifications of the GLMM models, for correlated connectivity indices**

Alternative scenarios were examined to assess the robustness of our results in the face of the high degree of correlation between the connectivity indices. As discussed in the main text, GDP and epidemic vulnerability displayed high correlations amongst themselves (Spearman’s ƿ = 0.88, p <.001). We consider scenarios where either connectivity index was included in separate models. In both instances we consider the goodness of fit, using the calculated marginal GLMM R2, to estimate the proportion of variation explained by the fixed effects and the resultant difference in their fixed effects Akaike information criteria (AIC).

Comparing both scenarios, most of the estimated coefficients (and odds ratio) of the connectivity indices are broadly stable and similar. However, the comparative fit test shows that the model with epidemic vulnerability had a slightly better fit than the model with GDP (S1 Table).

**S1 Table. Comparative-fit test for GLMM model including epidemic vulnerability and GLMM model including GDP**.

|  | **Model with epidemic vulnerability** | |  | **Model with GDP** | |  |
| --- | --- | --- | --- | --- | --- | --- |
|  | Coefficient  [95% CI] | Odd ratio  [95% CI] | | Coefficient  [95% CI] | Odd ratio  [95% CI] | |
| **Connectivity Index** |  |  | |  |  | |
| Dengue activity | 0.31  [0.18, 0.44] | 1.36  [1.19, 1.55] | | 0.30  [0.17, 0.43] | 1.35  [1.19, 1.54] | |
| Dengue seasonality | 0.12  [-0.01, 0.25] | 1.13  [0.99, 1.29] | | 0.14  [0.01, 0.27] | 1.15  [1.01, 1.31] | |
| Incidence estimates | 1.44  [1.33, 1.55] | 4.23  [3.79, 4.72] | | 1.41  [1.29, 1.52] | 4.08  [3.65, 4.56] | |
| Geographical distance | -0.52  [-0.65, -0.38] | 0.60  [0.52, 0.69] | | -0.55  [-0.69, -0.42] | 0.58  [0.50, 0.66] | |
| Total Air passengers | 2.01  [1.90, 2.13] | 7.49  [6.68, 8.39] | | 2.03  [1.92, 2.14] | 7.61  [6.81, 8.49] | |
| Epidemic vulnerability | 0.19  [0.10, 0.27] | 1.20  [1.10, 1.32] | |  |  | |
| GDP |  |  | | 0.26  [0.18, 0.33] | 1.29  [1.20, 1.40] | |
| **Model Fit** |  |  | |  |  | |
| $\boldsymbol{R}_{\mathbf{GLMM}(\boldsymbol{m})}^{\boldsymbol{2}}$ | 0.507 | | | 0.502 | | |
| $\boldsymbol{R}_{\mathbf{GLMM}(\boldsymbol{c})}^{\boldsymbol{2}}$ | 0.656 | | | 0.650 | | |
| Fixed effects $\Delta$AIC | 0.0 | | | 26.7 | | |

## **Alternative specifications of the GLMM models, using count data**

We did consider an alternative specification of our GLMM models, using count data (i.e. the actual imported case numbers), to explore any potential loss of information in the transformation of our response variable to a binary one. To do this, we first ran the preliminary analysis using a Poisson model on the count data to determine the prima facie dispersion status of the model and subsequently ran a negative binomial model, with log link functions. After which we ran the binomial model with logit link function, with the response variable as a binary response. In all instance we consider the goodness of fit, using the calculated marginal GLMM R^2^, to estimate the proportion of variation explained by the fixed effects.

We did a comparative-fit test for both models (i.e. the count-data and the binary response), with the basic assumptions that either will lead to similar results in terms of estimated coefficient and their respective incidence rate ratio or odds ratio. The results of the negative binomial model, with log link functions (S2 Table), was similar to that of the binomial model with logit link function (reported in Table 3 of the main text) in terms of the strength, direction and statistical significance of the estimated coefficients.

However, the marginal GLMM R^2^, of the binomial model with a logit link function was higher, indicating a better fit for the fixed effects (S3 Table). So, contrary to the transformation of the response variable captured more of the variation of the fixed effects as opposing a loss of information. It is also worth mentioning the logit link function had better convergence properties in comparison to the log link. Hence, we opted for the GLMM logit link function, as best fitting to our data structure.

Below is a table with the comparative-fit test for both modeling approaches, likewise we have included a table with the estimated coefficient and incidence rate ratio for the count-data modeling approach to show the similarities with the logit-link results. If necessary, we can include this in the supporting information.

**S2 Table. Estimated coefficient and Incidence rate ratio for GLMM log link model (i.e. count data)**

|  | **Model 1** | |  | **Model 2** | |  | **Model 3** | |  |
| --- | --- | --- | --- | --- | --- | --- | --- | --- | --- |
|  | Coefficient [95% CI] | Incidence rate ratio  [95% CI] | | Coefficient [95% CI] | Incidence rate ratio  [95% CI] | | Coefficient  [95% CI] | Incidence rate ratio  [95% CI] | |
| **Connectivity Index** |  |  | |  |  | |  |  | |
| Dengue activity | 0.36  [0.22, 0.50] | 1.44  [1.25, 1.65] | | 0.30  [0.16, 0.44] | 1.35  [1.18, 1.56] | | 0.28  [0.13, 0.40] | 1.32  [1.15, 1.52] | |
| Dengue seasonality | -0.33  [-0.01, 0.25] | 0.97  [0.85, 1.11] | | -0.06  [-0.20, 0.08] | 0.94  [0.82, 1.08] | | -0.14  [-0.27, 0.00] | 0.87  [0.76, 1.00] | |
| Incidence estimates | 1.49  [1.38, 1.61] | 4.46  [3.99, 4.98] | | 1.97  [1.83, 2.11] | 7.17  [6.22, 8.27] | | 2.01  [1.87, 2.15] | 7.44  [6.48,8.55] | |
| Geographical distance | -0.33  [-0.45, -0.20] | 0.72  [0.64, 0.82] | | -0.93  [-1.08, -0.78] | 0.39  [0.34, 0.46] | | -1.09  [-1.25, -0.94] | 0.34  [0.29, 0.39] | |
| Epidemic vulnerability | 0.10  [0.01, 0.19] | 1.11  [1.01, 1.21] | | 0.39  [0.29, 0.48] | 1.47  [1.34, 1.62] | | 0.46  [0.37, 0.56] | 1.59  [1.44, 1.74] | |
| Total Air passengers | 2.31  [2.20, 2.43] | 10.09  [8.99, 11.32] | | 2.31  [2.17, 2.46] | 10.12  [8.74, 11.71] | | 2.00  [1.83, 2.18] | 7.41  [6.23, 8.82] | |
| **Centrality Measures** |  |  | |  |  | |  |  | |
| Degree | — | — | | — | — | | 0.54  [0.37, 0.72] | 1.72  [1.45, 2.05] | |
| Betweenness | — | — | | — | — | | 1.09  [0.95, 1.23] | 2.96  [2.57, 3.42] | |
| Closeness | — | — | | 0.14  [0.01, 0.27] | 1.15  [1.01, 1.30] | | — | — | |
| Eigenvector | — | — | | 1.10  [0.95, 1.25] | 3.01  [2.59, 3.49] | | — | — | |
| **Model Fit** |  |  | |  |  | |  |  | |
| $\mathbf{R}_{\mathbf{GLMM}(\mathbf{m})}^{\mathbf{2}}$ | 0.443 | | | 0.492 | | | 0.490 | | |
| $\mathbf{R}_{\mathbf{GLMM}(\mathbf{c})}^{\mathbf{2}}$ | 0.589 | | | 0.625 | | | 0.619 | | |
| Fixed effects $\Delta$AIC | 269.7 | | | 59.1 | | | 0 | | |

**Model 1**: the base model of connectivity indices; **Model 2**: Base model with closeness and eigenvector centrality measures; **Model 3**: Base model with the degree and betweenness centrality measures. Log-link utilizes the count data, i.e. actual number of imported dengue cases; while the Logit-link utilizes a binary response variable, indicating an imported dengue case or not.

**S3 Table. General comparative-fit test for GLMM log-link (count data) & GLMM logit link (binary response) models.**

| **Model Fit** | **Model 1** | | **Model 2** | | **Model 3** | |
| --- | --- | --- | --- | --- | --- | --- |
|  | **Log-link** | **Logit-link** | **Log-link** | **Logit-link** | **Log-link** | **Logit-link** |
| $\mathbf{R}_{\mathbf{GLMM(m)}}^{\mathbf{2}}$ | 0.443 | 0.507 | 0.492 | 0.535 | 0.490 | 0.531 |
| $\mathbf{R}_{\mathbf{GLMM(c)}}^{\mathbf{2}}$ | 0.589 | 0.656 | 0.625 | 0.676 | 0.619 | 0.673 |

**Model 1**: the base model of connectivity indices; **Model 2**: Base model with closeness and eigenvector centrality measures; **Model 3**: Base model with the degree and betweenness centrality measures. Log-link utilizes the count data, i.e. actual number of imported dengue cases; while the Logit-link utilizes a binary response variable, indicating an imported dengue case or not.
